# A continuous repetitive task to detect fatigability in spinal muscular atrophy

**DOI:** 10.1186/s13023-018-0904-5

**Published:** 2018-09-12

**Authors:** Marloes Stam, Renske I. Wadman, Bart Bartels, Maureen Leeuw, Henk-Jan Westeneng, Camiel A. Wijngaarde, Leonard H. van den Berg, W. Ludo van der Pol

**Affiliations:** 1 Department of Neurology, Brain Center Rudolf Magnus Institute of Neuroscience, Utrecht University, University Medical Center Utrecht, Heidelberglaan 100, F02.230, 3508 GA Utrecht, The Netherlands; 2 Child Development and Exercise Center, Wilhelmina Children’s Hospital, Utrecht University, University Medical Center Utrecht, Lundlaan 6, 3584 EA Utrecht, The Netherlands

**Keywords:** Neuromuscular disease, Spinal muscular atrophy, SMA, Clinical neurology, Fatigability, Outcome measure, Repeated nine-hole peg test, r9HPT

## Abstract

**Background:**

To determine the value of a continuous repetitive task to detect and quantify fatigability as additional dimension of impaired motor function in patients with hereditary proximal spinal muscular atrophy (SMA).

**Results:**

In this repeated measure case-control study 52 patients with SMA types 2–4, 17 healthy and 29 disease controls performed five consecutive rounds of the Nine-Hole Peg test to determine the presence of fatigability. We analysed differences in test performance and associations with disease characteristics. Five patients with SMA type 2 (22%) and 1 disease control (3%) could not finish five rounds due to fatigue (*p* = 0.01). Patients with SMA type 2 performed the test significantly more slowly than all other groups (*p* < 0.005) and disease controls were slower than healthy controls (*p* < 0.05). Patients with SMA type 2 performed round five 27% slower than round one, while healthy controls performed round five 14% faster than round one (*p* = 0.005). There was no difference between SMA type 3a, type 3b/4 or disease controls and healthy controls (*p* > 0.4). Time needed to complete each round during the five-round task increased in 15 patients with SMA type 2 (65%), 4 with type 3a (36%), 4 with type 3b/4 (22%), 9 disease controls (31%) and 1 healthy control (6%). There was no effect of age at disease onset or disease duration in SMA type 2 (*p* = 0.39). Test-retest reliability was high.

**Conclusion:**

Fatigability of remaining arm function is a feature of SMA type 2 and can be determined with continuous repetitive tasks.

## Background

Spinal muscular atrophy (SMA), caused by homozygous deletion or disabling mutations of the survival motor neuron (*SMN*) 1 gene, [1] is one of the most common hereditary neuromuscular diseases [2]. Deficiency of SMN protein primarily, but not exclusively, [3, 4] affects lower motor neurons leading to muscle atrophy and weakness, with considerable variability in severity between patients [5, 6]. In addition to weakness, patients with SMA often mention a lack of stamina during daily activities. Patients experience fatigability during tasks of daily life such as prolonged (power)wheelchair driving, during eating when lifting cutlery repeatedly and when chewing food. Patients also report severe limitations in daily living, including social events, work and sports due to fatigue and fatigability, with a potential negative effect on quality of life. Observations during isometric muscle contraction endurance tests [7] and the six-minute walk test (6MWT) [8, 9] suggest that abnormal muscle fatigability (i.e. a decrease in performance over a given time or sustained measure of mechanical output [10]) represents an additional dimension of attenuated motor function in SMA. Causes of fatigability in SMA may be multiple, including altered muscle metabolism [4, 11] or abnormal neuromuscular junction (NMJ) anatomy and function [12–14]. With recent advances in therapy development, the need for relevant outcome measures has become more urgent [15]. Motor scales that are currently used in SMA research do not specifically measure muscle fatigability [16–18] and the assessment of fatigability using other tests has been inconsistent, [8, 9, 19, 20] underlining the fact that we need additional tools to determine the presence, extent and causes of fatigability. Tests that require repetitive muscle contractions may be most sensitive for determining fatigability in conditions characterized by NMJ disorders, possibly including SMA [12]. We, therefore, investigated the repeated nine-hole peg test (r9HPT) to determine fatigability in a repeated measure case-control study design.

## Methods

### Participants

Patients with SMA types 2, 3 and 4 were recruited from the Dutch SMA register (www.treat-nmd.eu/patientregistries) (i.e. symptom onset at age > 6 months, < 18 months; > 18 months, < 30 years; and > 30 years, respectively and highest acquired motor milestones: the ability to sit for SMA type 2 and the ability to walk independently for SMA types 3 and 4) [6]. An additional subdivision was made: type 3a with symptom onset > 18 months, but < 3 years and type 3b with symptom onset > 3 years and < 30 years [5]. In case of discrepancy between age at symptom onset and highest acquired motor milestone, the latter was used to define SMA type. To minimize selection bias, all eligible patients enrolled in this register were offered the possibility to participate. All patients had a homozygous deletion of the *SMN1* gene or a heterozygous *SMN1* deletion in combination with a point mutation on the second *SMN1* allele.

Disease controls were patients with other neuromuscular disorders who visited the pediatric and adult neuromuscular outpatient clinic of the University Medical Center Utrecht, the Netherlands. Healthy controls were recruited by participating SMA patients. All participants had to be over the age of 5 years. Additional exclusion criteria were a history of myasthenia gravis or other myasthenic syndromes or any other neuromuscular disorder known to affect NMJ function, or the use of pyridostigmine.

### The repeated nine-hole peg test (r9HPT)

Participants were asked to perform five rounds of the Nine-Hole Peg test [21–23] (r9HPT) with the Rolyan® 9HPT (Patterson Medical, Homecraft Rolyan; Sutton-in-Ashfield, United Kingdom). All patients were instructed to take 9 lightweight plastic pegs one by one from a container and place them in 9 holes on the board as fast as possible, then remove them one at a time and replace them in the container. They had to perform 5 consecutive rounds without a break, using the same, preferred hand. Participants were encouraged to complete the task as fast as possible. The time required to complete each round was recorded with a stopwatch. If participants dropped a peg, they continued with the task while we placed the peg back in the container. We also recorded all other events that might slow down test performance. The r9HPT was conducted at the outpatient clinic or at the patient’s home using a height-adjusted table and chair, with both feet positioned on the floor, or on the table attached to the patient’s wheelchair. The participant supported the test board, using the non-performing hand. The container on the board could be positioned at the side of the participant’s choice. The r9HPT was conducted twice to assess test reproducibility. If it was performed twice on the same day, there was a resting period of at least 15 min between trials.

### Statistical analyses

We used a random intercept, random slope linear mixed model (LMM) to assess r9HPT performance between groups while accounting for inter-subject variance. Age and gender were added to the model as covariates. Subsequently we used the LMM to calculate the effects of age at symptom onset and disease duration on test performance in patients with SMA type 2. To evaluate incomplete test performance due to fatigue, we performed a Kaplan Meyer survival analysis, using the log-rank test to compare survival curves between groups. We evaluated incidents that might have slowed down test performance (e.g. dropping a peg). If the round time in which the incident occurred was (equal to) the slowest test measurement, the value was removed and treated as missing. We calculated the slope of the linear regression line through the five data points (i.e. seconds to perform each round) for each participant to identify participant characteristics in relation to test performance.

We assessed test reproducibility by computing two-way mixed intra-class correlation coefficients (ICC), type consistency, for each round and corresponding round of the first and second r9HPT trial. *p* values < 0.05 were significant. We used SPSS (IBM SPSS Statistics version 20;IBM Inc., Chicago, IL) and R (R version 3.2.0 (Full of ingredients); R Foundation for statistical computing, Vienna, Austria) for statistical analysis.

The sample size was not calculated prospectively, because of the exploratory nature of this study and unpredictable effect size. Sample size was determined by the number of eligible patients willing to participate.

## Results

### Patients

Ninety-eight participants performed the r9HPT, including fifty-two SMA patients (23 SMA type 2; 11 type 3a; 16 type 3b; 2 type 4), 17 healthy controls and 29 disease controls (11 Duchenne muscular dystrophy; 6 hereditary motor and sensory neuropathy (HMSN); 5 limb girdle muscular dystrophy (LGMD); 2 Becker congenital myotonia; 1 Becker muscular dystrophy; 1 Bethlem myopathy; 1 chronic inflammatory demyelinating polyneuropathy (CIDP); 1 progressive muscular atrophy (PMA); 1 suspected muscular dystrophy). Patient characteristics are summarized in Table 1**.**

**Table 1:** Baseline Characteristics

	SMA		SMA type 2	SMA type 3a	SMA type 3b/4	Healthy controls	Disease controls
	(*N* = 52)		(*N* = 23)	(*N* = 11)	(*N* = 18)	(*N* = 17)	(*N* = 29)
Gender, male	17 (33%)		5 (22%)	3 (27%)	9 (50%)	3 (18%)	23 (79%)
Age, y, median (range)^a^		28 (7–72)	18 (6–70)	29 (9–52)	41 (14–72)	33 (6–73)	13 (8–76)
Age symptom onset, m, median (range)		18 (4–456)	10 (4–30)	18 (8–24)	138 (42–456)	NA	21 (10–672)
Disease duration, y, median (range)^a^		22 (1–69)	17 (5–69)	29 (7–51)	24 (1–66)	NA	8 (1–20)
Ambulant, N (%)		16 (31%)	0	3 (27%)	13 (72%)	17 (100%)	23 (79%)
*SMN2* copy number	*3*	30	21	7	2	–	–
	*4*	20	1	3	16	–	–
	*U*	2	1	1	–	–	–
No. of r9HPT completed	*1×*	9	5	2	2	1	3
	*2×*	43	18	9	16	16	26
Time between 1st and 2nd r9HPT, d, median (range)		97 (0–696)	158 (0–696)	69 (0–249)	69 (0–407)	0 (0–253)	254 (0–469)
Mean speed, s/round, (range)^a^			45.6 (20–156)	22.1 (17–41)	18.8 (18–35)	16.8 (14–26)	29.2 (18–113)

*Abbreviations*: *SMN* survival motor neuron, *U* unknown, *N* number, *NA* not applicable, *y* years, *m* months, *d* days, *s* seconds, *r9HPT* repeated nine hole peg test, *no.* number

^a^values at first performed r9HPT

### Repeated nine-hole peg test

**Fig. 1:**
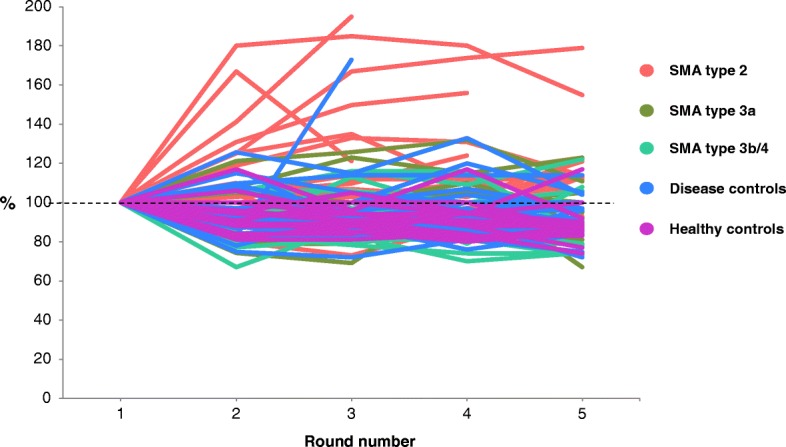
Results of the repeated 9HPT in patients with SMA and (disease) controls. The time needed to complete the first round was set as 100%; subsequent rounds are expressed as percentages compared to baseline

**Fig. 2:**
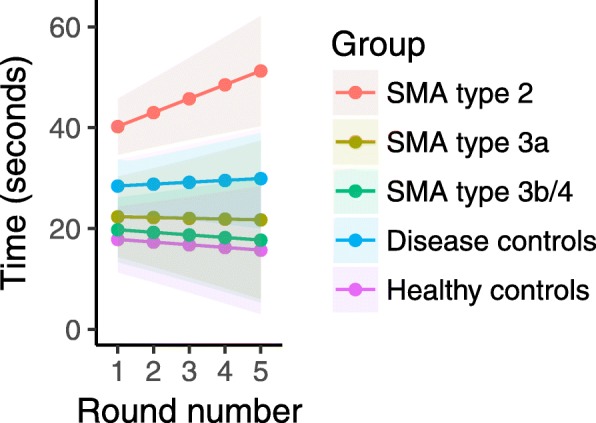
Mean repeated 9HPT results for each participant group. Mean time needed (seconds) to complete each round for each participant group and 95% confidence intervals

**Fig. 3:**
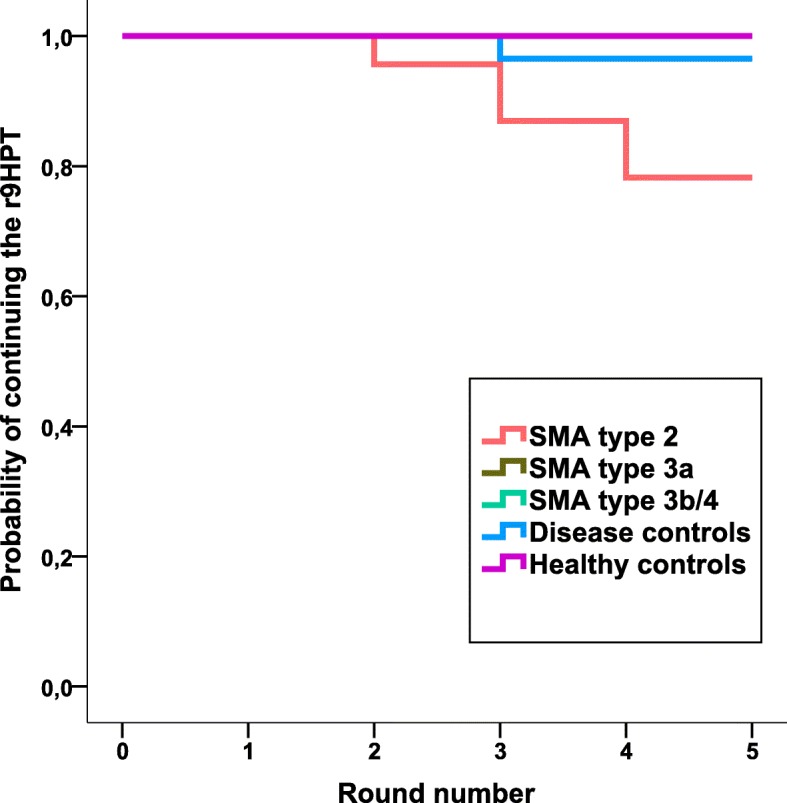
Participants with uncompleted repeated 9HPT due to exhaustion. Kaplan Meyer curves depicting round numbers at which subjects had to stop due to exhaustion

Results are summarized in Fig. 1. Six participants (2 patients with SMA type 3b, 1 healthy control and 3 disease controls) dropped a peg during one of the five rounds, which resulted in a slower time for that round. As described, these values were treated as missing in the analyses. Mean speed at which the test was performed (seconds per round (sec/round)) was 45.6 s/round (95% CI 37.5–53.7 s/round) for SMA type 2 patients, 22.1 s/round (95% CI 10.4–33.7 s/round) for SMA type 3a patients, 18.8 s/round (95% CI 9.6–28.0 s/round) for SMA type 3b/4 patients, 29.2 s/round (95% CI 21.7–35.6 s/round) for disease controls and 16.8 s/round (95% CI 7.4–26.2 s/round) for healthy controls. Mean speed was significantly slower in SMA type 2 patients (*p* < 0.001) and disease controls than in healthy controls (*p* < 0.05). There was no difference in mean speed between healthy controls and SMA type 3a patients (*p* = 0.3) or SMA type 3b/4 patients (*p* = 0.6). Compared to disease controls, SMA type 2 patients were significantly slower (*p* < 0.005) **(**Fig. 2**)**. When looking at the interaction effect of participant groups and round number to evaluate test performance during 5 subsequent rounds, there was a significant difference between patients with SMA type 2 and healthy controls (*p* = 0.005), but not between other patient groups (SMA type 3a, SMA type 3b/4, disease controls) and healthy controls (*p* > 0.4): SMA type 2 patients performed round five (51.3 s, 95% CI: 40.3–62.2 s) 27.4% slower than round one (40.2 s, 95% CI: 34.6–45.8 s), SMA type 3a patients performed round five (21.7 s, 95% CI: 6–37.5 s) 2.8% faster than round one (22.4 s, 95% CI: 14.4–30.3 s), SMA type 3b/4 patients performed round five (17.7 s, 95% CI: 5.3–30.1 s) 11.6% faster than round one (19.8 s, 95% CI: 13.4–26.2 s), disease controls performed round five (29.9 s, 20.0–39.8 s) 5.2% slower than round one (28.4 s, 95% CI: 23.2–33.7 s) and healthy controls performed round five (15.7 s, 95% CI: 3.0–28.4 s) 13.5% faster than round one (17.9 s, 95% CI: 11.3–24.4 s) **(**Fig. 2**)**. Neither age at disease onset nor disease duration influenced test performance in patients with SMA type 2 (*p* = 0.4 and *p* = 0.7). Based on the slope of their individual linear regression lines, the time needed to complete each round during the five-round task increased in 15 patients with SMA type 2 (65%), 4 patients with SMA type 3a (36%), 4 patients with SMA type 3b/4 (22%), 9 disease controls (31%) and 1 healthy control (6%). Characteristics of these participants are summarized in Table 2, sorted by the magnitude of the slope during five rounds**.** These include five patients with SMA type 2 (22%) and one disease control (3%) who could not complete the test due to fatigue (*p* = 0.01) **(**Fig. 3**)**.

**Table 2:** Characteristics of participants with increased time needed to complete one round during the 5-round task

Gender	Age (y)	Disease	Disease duration (y)	ambulation	Slope^a^
Male	16.92	Duchenne	15.42	non-ambulant	34.5
Female	30.25	SMA type 2	29.50	non-ambulant	21.8
Male	23.75	SMA type 2	22.83	non-ambulant	21.6
Male	7.68	SMA type 2	7.39	non-ambulant	9.9
Female	7.69	SMA type 2	7.19	non-ambulant	6.0
Male	13.49	SMA type 2	12.66	non-ambulant	5.0
Female	70.28	SMA type 2	69.37	non-ambulant	3.2
Female	21.40	SMA type 2	21.06	non-ambulant	2.2
Male	9.54	SMA type 2	8.74	non-ambulant	1.6
Male	48.59	SMA type 3b/4	44.09	non-ambulant	1.5
Female	25.94	SMA type 2	25.52	non-ambulant	1.4
Female	38.77	SMA type 3a	37.61	non-ambulant	1.4
Female	24.41	SMA type 2	22.91	non-ambulant	1.2
Female	5.69	SMA type 2	4.61	non-ambulant	1.1
Male	75.70	PMA	19.70	ambulant	0.8
Male	9.56	Becker myotonia	8.14	ambulant	0.7
Female	37.98	SMA type 2	37.44	non-ambulant	0.6
Female	9.95	SMA type 3a	8.29	non-ambulant	0.6
Female	5.69	SMA type 2	4.69	non-ambulant	0.5
Female	29.51	Healthy control	na	ambulant	0.5
Male	38.00	SMA type 3b/4	13.50	ambulant	0.4
Male	13.33	Duchenne	u	non-ambulant	0.4
Female	13.64	SMA type 2	12.93	non-ambulant	0.4
Male	8.92	SMA type 3a	7.33	ambulant	0.2
Male	21.98	SMA type 3b/4	u	ambulant	0.2
Female	12.08	SMA type 2	11.33	non-ambulant	0.2
Female	21.83	SMA type 3a	20.42	non-ambulant	0.2
Female	7.73	HMSN	6.40	ambulant	0.2
Male	13.34	Duchenne	11.84	non-ambulant	0.1
Male	34.86	SMA type 3b/4	31.36	ambulant	0.1
Male	8.41	Duchenne	u	ambulant	0.1
Male	14.62	Duchenne	u	ambulant	0.1
Female	15.25	HMSN	13.92	ambulant	0.1

*Abbreviations*: *SMA* spinal muscular atrophy, *HMSN* hereditary motor and sensory neuropathy, *PMA* progressive muscular atrophy, *y* years, *u* unknown, *na* not applicable

^a^Slope: seconds increase per round

ICCs showed a high degree of test-retest reliability. The single measure ICCs for rounds 1–5 were (95%CI) 0.91 (0.86–0.94); 0.71 (0.58–0.73); 0.79 (0.68–0.86); 0.82 (0.72–0.88) and 0.88 (0.81–0.92), respectively.

## Discussion

Fatigability during activities of daily life may further incapacitate SMA patients who already experience disability due to muscle weakness. Time needed to complete rounds 2–5 of the r9HPT increased each round in patients with SMA type 2, indicating a reduced capacity to sustain a simple activity that mimics hand function in daily life.

Fatigability was previously suggested in ambulant SMA patients in a study that compared results of the first and sixth minute of the six-minute walk test (6MWT) [8, 9]. Additionally, the presence of fatigability in the upper limbs was implicated by an abnormal decremental response to repetitive nerve stimulation in 49% of patients with SMA types 2 and 3 [12]. Assessment of maximal isometric muscle contraction of both upper and lower limbs has failed to detect fatigability in SMA [20]. However, repetitive muscle contraction may be more sensitive than sustained isometric muscle contraction in assessing fatigability in disorders characterized by neuromuscular junction dysfunction, [24] including patients with SMA types 2 and 3 [12]. In this study we were able to document fatigability in the upper limbs, utilizing this concept, with a simple clinical test. The nine-hole peg test, originally designed to assess finger dexterity, [21–23] is cheap and we could reproduce the previously reported high test-retest reliability [25] in patients with SMA. Furthermore, severely affected SMA patients with minimal arm function can lift the lightweight pegs, but a possible disadvantage of the r9HPT is the clear ceiling effect that would limit its use to patients with SMA type 2, as reflected by the fact that most patients with SMA types 3 and 4 performed subsequent rounds at a similar or higher speed (Fig. 2), with group results that were comparable to healthy controls. Nevertheless, even ambulant patients with SMA type 3 often mention fatigability in connection with activities such as walking up/down stairs, and the results of this study provide proof of concept for the development of additional repetitive tests that could be tailored to the individual’s remaining motor function. The r9HPT could be improved by individually standardized test speed and although we do not consider it likely that fatigability in SMA type 2 is the result of a lack of motivation, since all patients visibly performed to the maximum of their abilities, we think that future studies should include questionnaires to determine motivation and pain during tests. Monitoring of heart rate and muscle recruitment by means of surface electromyography would be additional improvements to the current study protocol. Moreover, it is likely that slight modification of other existing tests that mimic arm and hand function in daily life activities, such as the Functional dexterity test [26] or the Box and Block test, [27] or of selected items of the Jebsen-Taylor hand function test, [28, 29] Motor Function Measure (MFM) [18] and Upper Limb Module [30] would be sufficient to yield a series of repeated-measure tests. Outcome measures for upper limb function that already employ repetitive flexion/extension movements of wrist and fingers (MoviPlate) and that have been validated in patients with Duchenne and used preliminary in SMA patients, [31, 32] could probably be adapted even more easily to measure fatigability.

Muscle weakness may play an important role in fatigue and fatigability. A previous study showed physiological fatigability in both healthy controls and patients with neuromuscular disorders during sustained maximal voluntary muscle contraction, [33] which indicates that fatigability is not only secondary to weakness. Since we did not document muscle strength of disease controls, we cannot exclude baseline differences between disease controls and patients with SMA type 2. There was, however, no effect of age at disease onset and disease duration, both surrogate markers for disease severity, on test performance in SMA type 2 patients. Moreover, we did not observe slowing of 9HPT performance in the majority of patients with SMA type 3a, despite the fact that many had significantly reduced muscle strength. These observations imply that fatigability in SMA type 2 is, at least partially, a separate dimension next to muscle weakness in SMA.

## Conclusion

We show that a simple continuous repetitive hand task provokes fatigability in patients with SMA type 2. Our results indicate that fatigability may represent an important dimension of reduced motor function, in addition to weakness, and that outcome measures of repetitive tasks could be used to document its presence. Developing tailored measures to quantify fatigability, implementable in clinical trials, could be an important step towards the development of (add-on) medication to treat fatigability in SMA, improving quality of life. Simple tests of repetitive muscle contractions that mimic important functions of daily life are a promising addition to existing outcome measures.

## Abbreviations

6MWT: Six-Minute Walk Test
CIDP: Chronic inflammatory demyelinating polyneuropathy
HMSN: Hereditary motor and sensory neuropathy
ICC: Intra-class correlation coefficients
LGMD: Limb girdle muscular dystrophy
LMM: Linear mixed model
MFM: Motor function measure
NMJ: Neuromuscular junction
PMA: Progressive muscular atrophy
r9HPT: Repeated Nine-Hole Peg Test
SMA: Spinal muscular atrophy
SMN: Survival motor neuron
